# Design, Synthesis and Biological Evaluation of New 1, 4-Dihydropyridine (DHP) Derivatives as Selective Cyclooxygenase-2 Inhibitors

**Published:** 2015

**Authors:** Iman Sabakhi, Vigen Topuzyan, Zahra Hajimahdi, Bahram Daraei, Hadi Arefi, Afshin Zarghi

**Affiliations:** a *Department of Medicinal Chemistry, School of Pharmacy, Shahid Beheshti University of Medical sciences, Tehran, Iran. *; b *The Scientific Thechnological Centre of Organic and Pharmaceutical Chemistry NASRAAL. Mnjoyan Institute of Fine Organic Chemistry, Yerevan, Armenia. *; c *Department of Toxicology, Faculty of Medical Sciences, Tarbiat Modares University, Tehran, Iran.*

**Keywords:** Synthesis, 1, 4-Dihydropyridine (DHP) Derivatives, COX-2 Inhibitors, Molecular modeling

## Abstract

As a continuous research for discovery of new COX-2 inhibitors, chemical synthesis, in vitro biological activity and molecular docking study of a new group of 1, 4-dihydropyridine (DHP) derivatives were presented. Novel synthesized compounds possessing a COX-2 SO_2_Me pharmacophore at the *para* position of C-4 phenyl ring, different hydrophobic groups (R_1_) at C-2 position and alkoxycarbonyl groups (COOR_2_) at C-3 position of 1, 4-dihydropyridine, displayed selective inhibitory activity against COX-2 isozyme. Among them, compound 5e was identified as the most potent and selective COX-2 inhibitor with IC_50_ value of 0.30 μM and COX-2 selectivity index of 92. Molecular docking study was performed to determine probable binding models of compound 5e. The study showed that the *p*-SO_2_Me-phenyl fragment of 5e inserted inside secondary COX-2 binding site (Arg^513^, Phe^518^, Gly^519^, and His^90^). The structure-activity relationships acquired reveal that compound 5e with methyl and ethoxycarbonyl as R_1_ and COOR_2_ substitutions has the necessary geometry to provide selective inhibition of the COX-2 isozyme and it can be a good basis for the development of new hits.

## Introduction

**Figure 1.**
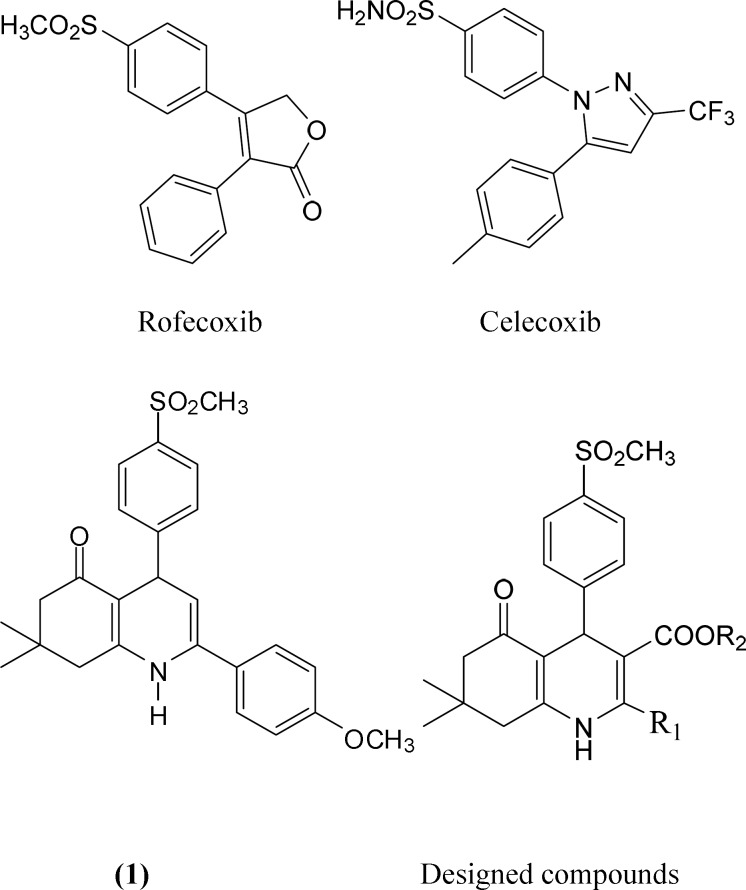
Selective COX-2 inhibitors (Rofecoxib, Celecoxib), lead compound (1) and designed scaffold.

Cyclooxygenase (COX) also known as prostaglandin synthase (PGH) is apotent mediator of inflammation. Non-steroidal anti-inflammatory drugs (NSAIDs) bind to cyclooxygenase, thereby inhibiting the production of prostaglandins. However, inhibition of COXs may lead to undesirable side effects. Nowadays, it is well established that there are at least two COX isozymes, COX-1 and COX-2 (1).The constitutive COX-1 isozyme is produced in a variety of tissues and appears to be important to the maintenance of physiological functions such as gastric protection and vascular homeostasis (2, 3). As COX-2 is usually specific to inflamed tissue, there is much less gastric irritation associated with COX-2 inhibition. This has led intense efforts in searching for potent and selective COX-2 inhibitors which could provide anti-inflammatory drugs with fewer risks. Several classes of compounds having selective COX-2inhibitory activity have been reported in the literature such as rofecoxib and celecoxib (Figure 1). Selective cyclooxygenase-2 (COX-2) inhibitors frequently belong to a class of diaryl heterocycles that possess two vicinal rings attached to a central heterocyclic scaffold in conjunction with a COX-2 pharmacophore such as a *para*-SO_2_Me substituent on one of the rings (4-6). As an initial attempt to discover novel COX-2 inhibitor with selectivity and safety profile, we have recently reportedseveral investigations describing the design, synthesis, and a molecular modeling study for a group of 5-oxo-1,4,5,6,7,8-hexahydroquinoline regioisomers including compound (1) from our compound library showed a good COX-2 inhibitory activity (Figure 1) (7).In continuation of our ongoing research work directed towards the development of selective COX-2 inhibitors, we have focused on the modification of compound (1) and designed some novel 1,4-dihydropyridines possessing *p*-SO_2_Me-phenyl moiety at C-4 position, different hydrophobic groups at C-2 position (R_1_) and different alkoxycarbonyl (COOR_2_) groups at the C-3 position (Figure 1).1,4-Dihydropyridines (DHP) are biologically and synthetically important class of compounds in the field of drugs and pharmaceuticals and have attracted attention of synthetic chemists due to their pharmacological properties (8, 9).The Hantzsch reaction is a well-known method for synthesizing of dihydropyridines (10). Hantzsch reaction is a kind of multi component reactions (MCRs) which have gained wide applicability in the field of synthetic organic chemistry as they increase the efficiency of the reaction and decrease the number of laboratory operations along with quantities of solvent and chemicals (11, 12).

In this study novel 1, 4-dihydropyridine derivatives were prepared according to Hantzsch reaction and evaluated for in vitro COX-1/COX-2 isozyme inhibition. We also performed docking studies to determine the orientation of the synthesized compounds in the COX-2 active site which led to the better understanding of the structure-activity relationship in designed COX-2 inhibitors.

## Experimental 

*General*

All chemicals and solvents used in this study were purchased from Merck AG and Aldrich Chemical. Melting points were determined using a Thomas-Hoover capillary apparatus. Infrared spectra were acquired using a Perkin Elmer Model 550 SE spectrometer. A Bruker AM-300 NMR spectrometer was used to acquire ^1^H NMR spectra with TMS as internal standard. Coupling constant (*J*) values are estimated in hertz (Hz) and spin multiples are given as s (singlet), d (double), t (triplet), q (quartet), m (multiplet), and br (broad). Low-resolution mass spectra were acquired with an MAT CH5/DF (Finnigan) mass spectrometer that was coupled on line to a Data General DS 50 data system. Electron-impact ionization was performed at an ionizing energy of 70 eV with a source temperature of 250^o^C. Elemental microanalyses, determined for C and H, were within ±0.4% of theoretical values. All chemicals and solvents used in this study were purchased from Merck AG and Aldrich Chemical. Melting points were determined with a Thomas-Hoover capillary apparatus. Infrared spectra were acquired using a Perkin Elmer Model 1420 spectrometer. A Bruker FT-500 MHz instrument (Bruker Biosciences, USA) was used to acquire ^1^HNMR spectra with TMS as internal standard. Chloroform-D was used as solvents. Coupling constant (*J*) values are estimated in hertz (Hz) and spin multiples are given as s (singlet), d (double), t (triplet), q (quartet), m (multiplet) and br (broad). The mass spectral measurements were performed on a 6410 Agilent LCMS triple quadrupole mass spectrometer (LCMS) with an electrospray ionization (ESI) interface.

*Chemistry*

**Scheme1:**
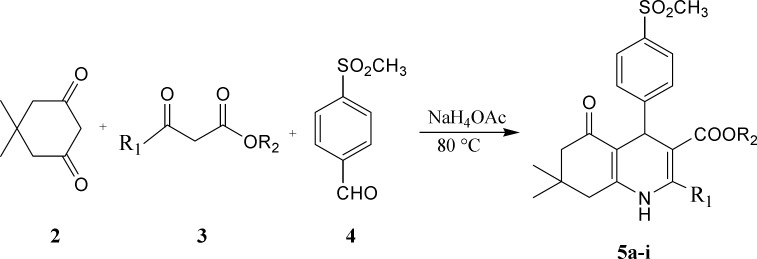
Synthesis of 1, 4-dihydropyridine derivatives (5a-i).

Preparation of1, 4-dihydropyridine derivatives based on Hantzsch method is shown in Scheme 1.Accordingly, a mixture of 5, 5-dimethyl-1,3-cyclohexandione (2), appropriate β-oxoesters (3) and 4-(methylsulfonyl)benzaldehyde (4) in the presence of ammonium acetate was refluxed in methanol to obtain target compounds (5a-i) in 54-95% yield.The structure of the synthesized compounds was confirmed by IR, ^1^H NMR and ESI-MS.

*General procedure for the synthesis of 1, 4-dihydropyridine derivatives* *(5a-i)*

A mixture of β-oxoesters (1 mmol), 4, 4-(5, 5)-dimethyl-1,3-cyclohexandione (1 mmol) and 4-(methylsulfonyl)benzaldehyde (1 mmol) in the presence of ammonium acetate (4 mmol) was refluxed in methanol at 80°C for overnight. After completion of the reaction, the mixture was cooled to room temperature; ethanol (10 mL) was added to dilute mixture. The mixture was poured into 80 mL ice water the precipitate was filtered off and washed with water. The crude products were purified by recrystallization from ethanol to give final products.

*Methyl-1, 4, 5, 6, 7, 8-hexahydro-2, 7, 7-trimethyl-4-(4-(methylsulfonyl)phenyl)-5-oxoquinoline-3-carboxylate (5a)*

Yield, 78%; mp: 244-245 °C; IR (KBr disk) υ (cm^-1^): 1150, 1300 (SO_2_); 1400-1600 (aromatic); 1689(C=O); 3356 (NH); ^1^HNMR (CDCl_3_, 500 MHz): δ 0.91 (s, 3H, CH_3_), 1.12 (s, 3H, CH_3_), 2.16-2.20 (m, 2H, dihydroquinoline H_8_), 2.26-2.29 (m, 2H, dihydroquinoline H_6_,* J=*15.8 Hz), 2.45 (s, 3H, CH_3_), 3.04 (s, 3H, SO_2_Me), 3.64 (s, 3H, CO_2_CH_3_), 5.17 (s, 1H, dihydroquinoline H_4_), 5.84 (s, 1H, NH), 7.54 (d, 2H, methanesulfonyl phenyl H_2'_& H_6'_, *J=*7.6 Hz), 7.81 (d, 2H,methanesulfonyl phenyl H_3'_& H_5'_, *J=*7.6 Hz); LC-MS (ESI)*m/z*: 404.3 (M+1, 100); Anal. Calcd. for C_21_H_25_NO_5_S: C, 62.51; H, 6.25; N, 3.47. Found: C, 62.81; H, 6.45; N, 3.59.

*Methyl-2-amino-1, 4, 5, 6, 7, 8-hexahydro-7, 7-dimethyl-4-(4-(methylsulfonyl)phenyl)-5-oxo quinolone-3-carboxylate (5b)*

Yield,64%; mp: 177-179°C; IR (KBr disk) υ (cm^-1^): 1150, 1300 (SO_2_); 1400-1600 (aromatic); 1697 (C = O); 3350 (NH);^1^HNMR (CDCl_3_, 500 MHz): δ 0.99 (s, 3H, CH_3_), 1.12 (s, 3H, CH_3_), 2.20 (d, 1H, dihydroquinoline H_8_*, J=*16.3 Hz), 2.29 (d, 1H, dihydroquinoline H_8_,* J = *16.3 Hz), 2.48 (s, 2H, dihydroquinoline H_6_), 3.07 (s, 3H, SO_2_Me), 3.62 (s, 3H, CO_2_CH_3_), 4.81 (s, 1H, NH), 6.27 (br s, 2H, NH_2_), 7.49 (d, 2H, methanesulfonyl phenyl H_2'_& H_6'_, *J=*8.2 Hz), 7.86 (d, 2H, methanesulfonyl phenyl H_3'_& H_5'_,* J=*8.2 Hz); LC-MS (ESI)*m/z*: 405.1 (M+1, 100); Anal. Calcd. for C_20_H_24_N_2_O_5_S: C, 59.39; H, 5.98; N, 6.93. Found: C, 59.71; H, 6.25; N, 6.59.

*Methyl-2-ethyl-1, 4, 5, 6, 7, 8-hexahydro-7,7-dimethyl-4-(4-(methylsulfonyl)phenyl)-5-oxoquinoline-3-carboxylate (5c)*

Yield, 87%; mp: 186.5-188°C; IR (KBr disk) υ (cm^-1^): 1150, 1300 (SO_2_); 1400-1600 (aromatic); 1697 (C = O); 3369 (NH);^1^HNMR (CDCl_3_, 500 MHz): δ 0.94 (s, 3H, CH_3_), 1.07 (s, 3H, CH_3_), 1.36 (t, 3H, CH_3_), 2.17-2.44 (m, 4H, dihydroquinoline H_6_& H_8_), 2.85 (q, 2H, CH_2_), 3.04 (s, 3H, SO_2_Me), 3.64 (s, 3H, CO_2_CH_3_), 5.19 (s, 1H, dihydroquinoline H_4_), 5.80 (br s, 1H, NH), 7.51 (d, 2H, methanesulfonyl phenyl H_2'_& H_6'_, *J=*8.1 Hz), 7.81 (d, 2H, methanesulfonyl phenyl H_3'_& H_5'_,* J=*8.2 Hz); LC-MS (ESI)*m/z*: 418.4 (M+1, 100); Anal. Calcd. for C_22_H_27_NO_5_S: C, 63.29; H, 6.52; N, 3.35. Found: C, 62.91; H, 6.35; N, 3.50.

*Methyl-1, 4, 5, 6, 7, 8-hexahydro-2-isopropyl-7, 7-dimethyl-4-(4-(methylsulfonyl)5-oxoquinoline-3-carboxylate (5d)*

Yield, 54%; mp: 163-164°C; IR (KBr disk) υ (cm^-1^): 1150, 1300 (SO_2_); 1400-1600 (aromatic); 1657 (C=O); 3352 (NH); ^1^HNMR (CDCl_3_, 500 MHz): δ 0.92 (s, 3H, CH_3_), 1.11 (s, 3H, CH_3_), 1.21 (d, 3H, CH_3_, *J=*6.9 Hz*)*, 1.27 (d, 3H, CH_3_, *J=*7.0 Hz), 2.19 (d, 1H, dihydroquinoline H_8_*, J=*16.3 Hz),2.29 (m, 2H, dihydroquinoline H_8_& H_6_,* J=*15.0 Hz), 2.45 (d, 1H, dihydroquinoline H_6_, *J=*16.6 Hz), 3.04 (s, 3H, SO_2_Me), 3.63 (s, 3H, CO_2_CH_3_), 4.27 (m, 1H, CH), 5.19 (s, 1H, dihydroquinoline H_4_), 6.07 (s, 1H, NH), 7.50 (d, 2H, methanesulfonyl phenyl H_2'_& H_6'_, *J=*8.3 Hz), 7.80 (d, 2H, methanesulfonyl phenyl H_3'_& H_5' _,* J=*8.3 Hz); LC-MS (ESI)*m/z*: 432.2 (M+1, 100); Anal. Calcd. for C_23_H_29_SO_5_N: C, 64.01; H, 6.77; N, 3.25. Found: C, 64.21; H, 6.95; N, 3.19.

*Ethyl-1, 4, 5, 6, 7, 8-hexahydro-2, 7, 7-trimethyl-4-(4-(methylsulfonyl)phenyl)-5-oxoquinoline-3-carboxylate (5e)*

Yield,81%;mp: 180.7-182.3°C;IR (KBr disk) υ (cm^-1^): 1150, 1300 (SO_2_); 1400-1600(aromatic); 1685(C = O); 3359 (NH);^1^HNMR (CDCl_3_, 500 MHz): δ 0.91 (s, 3H, CH_3_), 1.10 (s, 3H, CH_3_), 1.24 (t, 3H, CH_3_), 2.12-2.15 (d, 1H, dihydroquinoline H_8_), 2.18-2.28 (m, 2H, dihydroquinoline H_8 _& H_6 _), 2.35-2.38 (d, 1H, dihydroquinoline H_6_),2.40 (s, 3H, CH_3_), 3.01 (s, 3H, SO_2_Me), 4.06 (m, 2H, CH_2_), 5.14 (s, 1H, dihydroquinoline H_4_), 7.09 (s, 1H, NH), 7.51 (d, 2H, methanesulfonyl phenyl H_2'_& H_6'_, *J=*8.0 Hz), 7.76 (d, 2H, methanesulfonyl phenyl H_3'_& H_5'_,*J=*8.0 Hz); LC-MS (ESI)*m/z*: 418.1 (M+1, 100); Anal. Calcd. for C_22_H_27_NO_5_S: C, 63.29; H, 6.52; N, 3.35. Found: C, 63.41; H, 6.75; N, 3.42.

*Ethyl-1, 4, 5, 6, 7, 8-hexahydro-7,7-dimethyl-4-(4-(methylsulfonyl)phenyl)-5-oxo-2-propylquinoline-3-carboxylate (5f)*

Yield,54%; mp: 163-164°C; IR (KBr disk) υ (cm^-1^): 1150, 1300 (SO_2_); 1400-1600 (aromatic); 1658(C = O); 3305 (NH);^1^HNMR (CDCl_3_, 500 MHz): δ 0.88 (s, 3H, CH_3_), 1.05 (t, 3H, CH_3_), 1.12 (s, 3H, CH_3_), 1.27 (t, 3H, CH_3_), 1.71 (m, 4H, 2CH_2_), 2.16-2.20 (d, 1H, dihydroquinoline H_8_,* J = *16.2 Hz),2.26-2.29 (m, 2H, dihydroquinoline H_6 _& H_8_), 2.39-2.42 (d, 1H, dihydroquinoline H_6_,* J=*16.0 Hz), 3.04 (s, 3H, SO_2_Me), 4.07 (m, 2H, CH_2_), 5.19 (s, 1H, dihydroquinoline H_4_), 5.93 (br s, 1H, NH), 7.52 (d, 2H, methanesulfonyl phenyl H_2'_& H_6'_, *J=*7.9 Hz), 7.80 (d, 2H, methanesulfonyl phenyl H_3'_& H_5'_,* J=*7.8 Hz); LC-MS (ESI)*m/z*: 446.2 (M+1, 100); Anal. Calcd. for C_24_H_31_NO_5_S: C, 64.69; H, 7.01; N, 3.14. Found: C, 63.89; H, 6.95; N, 3.32.

*Ethyl-1, 4, 5, 6, 7, 8-hexahydro-7,7-dimethyl-4-(4-(methylsulfonyl)phenyl)-5-oxo-2-phenylquinoline-3-carboxylate (5g)*

Yield,87%; mp: 187.9-189 °C; IR (KBr disk) υ (cm^-1^): 1150, 1300 (SO_2_); 1400-1600 (aromatic); 1687(C = O); 3344 (NH);^1^HNMR (CDCl_3_, 500 MHz): δ 0.89 (t, 3H, CH_3_), 0.97 (s, 3H, CH_3_), 1.14 (s, 3H, CH_3_), 2.19-2.32 (m, 3H, dihydroquinoline H_6 _& H_8_), 2.45-2.48 (d, 1H, dihydroquinoline H_6_,* J=*16.6 Hz), 3.03 (s, 3H, SO_2_Me), 3.86 (m, 2H, CH_2_), 5.28 (s, 1H, dihydroquinoline H_4_), 6.07 (s, 1H, NH), 7.36 (m, 2H, benzyl H_3_& H_4_), 7.45 (m, 3H, benzyl H_2_, H_5_& H_6_), 7.68 (d, 2H, methanesulfonyl phenyl H_2'_& H_6'_, *J=*7.6 Hz), 7.85 (d, 2H, methanesulfonyl phenyl H_3'_& H_5'_,* J=*7.5 Hz); LC-MS (ESI)*m/z*: 480.2 (M+1, 100); Anal. Calcd. for C_27_H_29_NO_5_S: C, 67.62; H, 6.09; N, 2.92. Found: C, 63.96; H, 6.25; N, 3.12.

*t-Butyl-1, 4, 5, 6, 7, 8-hexahydro-2,7,7-trimethyl-4-(4-methanesulfonyl-phenyl)-5-oxo-quinoline-3-carboxylate (5h)*

Yield,95%; mp: 163-164°C;IR (KBr disk) υ (cm^-1^): 1150, 1300 (SO_2_); 1400-1600 (aromatic); 1694 (C = O); 3300-3500 (NH); ^1^HNMR (CDCl_3_, 500 MHz): δ 0.95 (s, 3H,°C H_3_), 1.11 (s, 3H, CH_3_), 1.37 (s, 9H,CH_3_), 2.14 (d, 1H, dihydroquinoline H_8_*, J=*16.3 Hz), 2.26 (d, 1H, dihydroquinoline H_8_,* J=*15.9 Hz), 2.36-2.39 (d, 2H, dihydroquinoline H_6_), 2.41 (s, 3H, CH_3_), 3.03 (s, 3H, SO_2_Me), 5.10 (s, 1H, dihydroquinoline H_4 _), 5.91 (br s, 1H, NH), 7.54 (d, 2H, methanesulfonyl phenyl H_2'_& H_6'_, *J=*8.2 Hz), 7.81 (d, 2H, methanesulfonyl phenyl H_3'_& H_5'_, *J=*8.2 Hz); LC-MS (ESI)*m/z*: 446.2 (M+1, 100); Anal. Calcd. for C_24_H_31_NO_5_S: C, 64.69; H, 7.01; N, 3.14. Found: C, 64.89; H, 7.21; N, 3.22.

*Benzyl-1, 4, 5, 6, 7, 8-hexahydro-2, 7, 7-trimethyl-4-(4-(methylsulfonyl)phenyl)quinoline-3-carboxylate (5i)*

Yield,87%; mp: 136.9-138.9 °C;IR (KBr disk) υ (cm^-1^): 1150, 1300 (SO_2_); 1400-1600 (aromatic); 1694 (C=O); 3557 (NH);^1^HNMR (CDCl_3_, 500 MHz): δ 0.86 (s, 3H, CH_3_), 1.04 (s, 3H, CH_3_), 2.01-2.07 (d, 1H, dihydroquinoline H_8_*, J=*16.3 Hz)*,* 2.17-2.20 (d, 1H, dihydroquinoline H_8_,* J=*16.4 Hz), 2.20-2.36 (q, 2H, dihydroquinoline H_6_), 2.38 (s, 3H, CH_3_), 2.96 (s, 3H, SO_2_Me), 4.98 (s, 2H, CH_2_), 5.10 (s, 1H, dihydroquinoline H_4_), 7.11-7.12 (m, 2H, benzyl H_2_& H_6_), 7.26 (m, 3H, benzyl H_3_, H_4 _& H_5_), 7.41 (d, 2H, methanesulfonyl phenyl H_2'_& H_6'_, *J=*8.3 Hz), 7.67 (d, 2H, methanesulfonyl phenyl H_3'_& H_5'_,* J=*8.3 Hz); LC-MS (ESI)*m/z*: 480.2 (M+1, 100); Anal. Calcd. for C_27_H_29_NO_5_S: C, 67.62; H, 6.09; N, 2.92. Found: C, 67.32; H, 5.84; N, 3.02.

*Molecular Modeling*

The active compound was selected for docking studies which performed using Autodock software Version 4.0. The ligand molecule was constructed using the Chem Draw and was energy minimized for 1000 iterations reaching a convergence of 0.01 kcal/mol Å. The coordinates of the X-ray crystal structure of COX-2 enzyme was obtained from the RCSB Protein Data Bank (3NT1) and the protein structure was prepared for docking. First of all, co-crystallized ligand and all water molecules were removed from crystal protein. Polar hydrogens wereadded and non polar hydrogens were merged, finally Kallman unitedatom charge and atom type parameter was added to 3NT1. Grid map dimensions (20×20×20) were set surrounding activesite. Lamarckian genetic search algorithmwas employed anddocking run was set to 50. The aim of docking is to search for suitable binding configuration between the ligands and the rigid protein. These docked structures were very similar to the minimized structures provided initially. The quality of the docked structures was determined by measuring the intermolcular energy of the ligand-enzyme assembly (13).

## Result and Discussion

A group of 1,4-dihydropyridine derivativespossessing a MeSO_2_ at the *para-*position of the C-4 phenyl ring, alkyl groups(R_1_) at the C-2 position and alkyloxycarbonyl groups(COOR_2_) at the C-3 position were prepared and evaluated for their ability to inhibit COX-1 and COX-2 using chemiluminescent kit (Cayman chemical, MI, USA) according to our previously reported method (14). Potent and selective COX-2 inhibitor, celecoxib was used as a reference compound inthe COX activity assay. All experiments were carried out at leastthree times and the data of inhibitory effects were summarized in Table 1.

**Table 1:** *In-vitro* COX-1 and COX-2 enzyme inhibition data for compounds 5a-i.

**Compound **	**R**_1_	**R**_2_	**IC**_50_ **(** **μ** **M)** a		**COX-2** **S.I.** b
			**COX-1 COX-2**		
**5a**	CH_3_	CH_3_	30.2	0.48	62.9
**5b**	NH_2_	CH_3_	31.0	0.44	70.4
**5c**	CH_2_CH_3_	CH_3_	30.7	0.59	52.1
**5d**	CH(CH_3_)_2_	CH_3_	27.6	0.62	43.1
**5e**	CH_3_	CH_2_CH_3_	27.6	0.30	92.0
**5f**	CH_2_CH_2_CH_3_	CH_2_CH_3_	25.2	1.38	18.2
**5g**	C_6_H_5_	CH_2_CH_3_	46.1	>100	-
**5h**	CH_3_	C(CH_3_)_3_	25.5	0.40	63.7
**5i**	CH_3_	CH_2_C_6_H_5_	22.9	>100	-
celecoxib			24.3	0.06	405

a Values are mean values of two determinations acquired using an ovine COX-1/COX-2 assay kit, where the deviation from the mean is < 10% of the mean value.

b *In-vit*ro COX-2 selectivity index (COX-1 IC_50_/ COX-2 IC_50_).

As shown in Table 1, all compounds except 5i and 5g (IC_50 _> 100 μM) displayed moderate to good inhibitory activities against COX-2 and were more potent inhibitor of COX-2 (IC_50 _= 0.3-1.38 μM range) than COX-1 (IC_50 _= 22.9-46.1 M range) with COX-2 selectivity indexes (SI) inthe range of 18.2-92.0. However, in all cases, the measured activities were lower than that of celecoxib. Our results indicated that different hydrophobic substituents at C-2 and C-3 position of 1, 4-dihydropyridine core affected the activity of the target molecules. In compounds series possessing methoxycarbonyl as COOR_2_ group (5a-d), replacement of methyl (5a, IC_50_ = 0.48 μM) at C-2 position with other alkyl groups such as ethyl (5c, IC_50_ = 0.59 μM) and isopropyl (5d, IC_50_ =0.62 μM) slightly decreased the COX-2 inhibitory activity. Compound 5b showed approximately similar potency (IC_50_ = 0.44 μM) to compound 5a. This may be due to isosteric replacement of methyl group with NH_2_ group in compound 5b. It is found that replacement of methoxycarbonyl with ethoxycarbonyl as R_2_ group in compound 5a resulted in compound 5e with improved COX-2 inhibitory effect (IC_50_ = 0.30 μM). Introduction of larger groups such as propyl and phenyl at C-2 position of compound 5e led to compounds 5f and 5g with significant loss of activities. Theexperimental results showed that *t*-butoxycarbonyl as COOR_2_ group is well tolerated and the corresponding compound, 5h exhibited IC_50_ value of 0.40 M. In addition, modification of ethoxycarbonyl group to benzyloxycarbonyl group in compound 5e led to compound 5i with no activity (IC_50_>100 μM). This may be due to large size of substitution and resulting steric hindrance. The effects of substituents introduced into the 1, 4-dihydropyridines moiety of compounds demonstrated that methyl and ethoxycarbonyl groups were the most appropriate substitutions at C-2 and C-3 positions, respectively and the corresponding compound, 5e was the most potent COX-2 inhibitor in this series with IC_50_ value of 0.30 μM and COX-2 selectivity index of 92.

**Figure 2:**
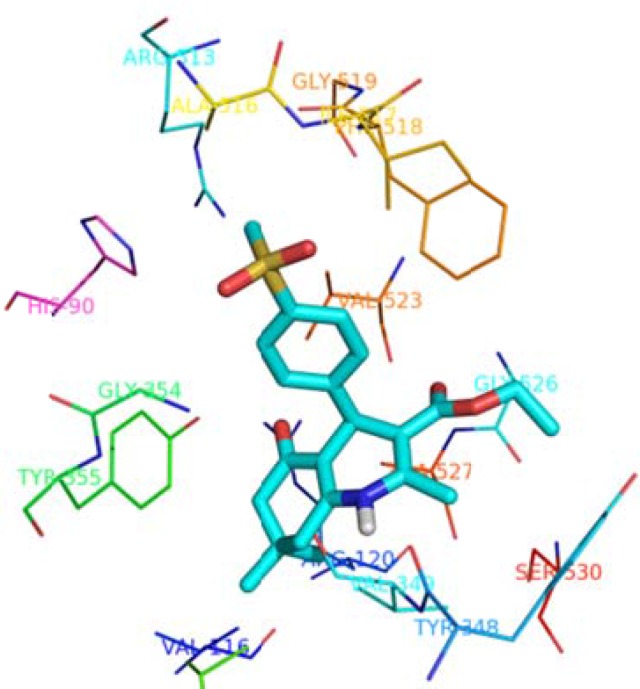
Binding mode of compound 5e in the COX-2 active site.

Molecular docking studies helpto understand the various interactions between the most active ligand (5e) and enzyme active sites in details. According to docking studies results (Figure 2), it is clear that *p*-SO_2_Me-phenyl moiety of compound 5e inserts deep inside the COX-2 active site pocket and forming hydrogen bond with Arg^513^ (distance = 4.8 Ǻ) and His^90^ (distance = 3.1 Ǻ). In addition, the N-*H*of the 1, 4-dihydropyridine scaffold interacts with C=O of Val^349 ^(distance = 4.0 Å). Moreover, the carbonyl group of central ring and ethoxycarbonyl bind to Arg^120^ (distance = 2.8 Å) and Gly^526^ (distance = 3.9 Å) through hydrogen bonds, respectively. Molecular docking studies associated with experimental results showed that compound 5e possesses the pharmacophoric requisites for COX-2 inhibition.

## Conclusion

In conclusion, new 1, 4-dihydropyridine derivatives were synthesized and evaluated for COX-1/COX-2 inhibition. Among them, compound 5e exhibited good COX-2 inhibitory activity and selectivity (IC_50_=0.30 μM and COX-2 selectivity index=92). Experimental results in conjunction with molecular docking studies indicated that compound 5e with methyl and ethoxycarbonyl groups as R_1_ and COOR_2_ substitutionscould interactappropriately with COX-2 active site. Therefore, this compound provides a promising lead for further development.
